# Rapid Demagnetization of New Hybrid Core for Energy Harvesting

**DOI:** 10.3390/s22062102

**Published:** 2022-03-09

**Authors:** Rafał Mech, Przemysław Wiewiórski, Karol Wachtarczyk

**Affiliations:** Faculty of Mechanical Engineering, Wroclaw University of Science and Technology, 50-370 Wroclaw, Poland; przemyslaw.wiewiorski@pwr.edu.pl (P.W.); karol.wachtarczyk@pwr.edu.pl (K.W.)

**Keywords:** SMART materials, magnetostriction, Terfenol-D, energy harvesting

## Abstract

This paper presents the results obtained using the rapid demagnetization method in the case of an NdFeB magnet and a new hybrid core. The developed core consists of three basic elements: an NdFeB magnet, Terfenol-D, and a specifically developed metallic alloy prepared by means of a suction casting method. The main goal of proposing a new type of core in the event of rapid demagnetization is to partially replace the permanent magnet with another material to reduce the rare-earth material while keeping the amount of generated electricity at a level that makes it possible to power low-power electrical devices. To “capture” the rapid change of magnetic flux, a small number of coils were made around the core. However, the very low voltage level at very high current required the use of specialized electronic transducers capable of delivering a voltage level appropriate for powering a microprocessor system. To overcome this problem, a circuit designed by the authors that enabled voltage processing from low impedance magnetic circuits was used. The obtained results demonstrated the usefulness of the system at resonant frequencies of up to 1 MHz.

## 1. Introduction

Interest in energy harvesting (EH) is continuously increasing from year to year. The primary impulse for development in the discussed area is related to the shortage of electricity and the search for new possible sources of energy. In this context, the idea of EH would also include the generation of energy from wind, solar plants, and other natural power sources on a large scale. In the literature, the discussion of energy harvesting is limited to small electrical and electronic devices that can operate in a self-sufficient manner [1,2,3]. On the basis of this limitation, it has become clear that EH cannot be considered as a feasible power source in high-power applications. However, as could be predicted, increasing the number of devices that can be powered from harvested energy would make it possible to relieve other energy sources [4,5,6,7,8]. Obtaining energy for devices from the environment has found possible applications in many different markets, and the number of possible applications will grow continuously [9,10,11,12,13]. Currently, the consumer device market is not large, but it is predicted that it will increase continuously, and will have an influence on the direction of future research. Important features and attributes have had, and still have, an influence on the directions of EH development and potential target markets, including:

- Small size, portability;
- Capability of performing in difficult-to-access environments;
- Fewer cables and reduced usage of other materials, devices, and systems;
- Provision of real-time information;
- Decreased cost;
- Low maintenance needs;
- Reduction of batteries—green energy;
- Great market potential, and the absorptiveness of the market.

Due to their small size and lack of need for complicated powering assemblies, these devices can be used in difficult-to-access environments [14,15,16], such as in complicated machines, structures, etc. Research on the use of EH devices in very fragile environments such as the human body was performed at Southampton University Hospital in the UK. A prototype of an electrodynamics harvester was created that was able to generate enough energy from heartbeats to supply power to a pacemaker for the entire life of a patient. The output was 4.3 micro joules per heartbeat; with the use of new and better polymer materials, that value is expected to double [1].

Additionally, self-sufficient devices mean less cable. This applies mainly to sensors, and is a very important advantage, making monitoring constructions, machines, and different types of elements much easier and safer to use, while providing better information for decision-making processes like with respect to the maintenance needs of an object [17]. These advantages were noticed by the American Society of Civil Engineers, which together with the University of Texas (UT), began testing a new wireless bridge monitoring system [18]. Another system for monitoring the condition of railway bridge structures was presented in Ref. [19]. Sensors that do not need a separate powering system make it possible to test bridges more quickly and receive more detailed information in real time. The importance of such kinds of systems is evidenced by the fact that in America in 2008, over 17,000 bridges did not meet the requirements necessary for operation.

The research and development on generating energy is progressing very quickly, opening new markets for a growing range of different devices. Consumers and the military, along with third-world markets, are the main groups, and will create the biggest market for devices by value. Their share accounted for around 88% of all value predicted for such kinds of device in 2014. As projects are run mainly for profit (including both present and future profits, both financial and strategic), it seems to be significant to realize the importance and the full potential of the EH concept and technology. According to the report presented by IDTechEx Ltd. (Cambridge, UK) [20], these potential markets could represent an income of about USD 1.156 trillion per year.

The main idea of the presented research was to determine whether the chosen types of harvesting device and the proposed type of excitation could be useable for mechanical purposes. The main goal of this investigation was to determine whether the new proposed hybrid core structure, when applied in dedicated harvesters, is able to provide sufficient energy to supply the chosen ATMEL microcontroller. The tests were carried out on a dedicated stand, allowing the impulse load to be applied with a fixed value. The results of the impulse investigations are discussed in detail to provide information about the usefulness of the proposed hybrid core in the area of energy harvesting.

## 2. Materials and Methods

The study presented in this paper was based on a well-known giant magnetostriction material, Terfenol-D, and on a material prepared by the authors, produced by the suction casting method. In addition, commercially available NdFeB neodymium magnets were also used in the investigation.

### 2.1. Suction Casting Alloy

The newly created material in the form of a metallic alloy described in this paper was developed based on literature data describing a patented soft, magnetic, amorphous material known as FINEMET [21,22]. It should be noted that the material with the composition Fe_57_Co_10_B_20_Si_5_Nb_4_V_4_ described in this paper was developed in the opposite direction to what has been the case in many studies showing materials based on FINEMET. The material presented in this work has a lower content of the basic elements, such as Fe and Co. It should also be emphasized that when describing the material, its composition is given in atomic notation, not mass notation. An arc furnace (arc-melter) was used to produce the material. To ensure the highest possible representation of the developed chemical composition of the alloy, very high-purity elements—up to 99.999%—were used for its production. Each of the alloying elements was weighed out to 4 decimal places, and then these components were placed in the arc furnace chamber. Next, the furnace chamber was sealed, and then the air was removed from it by pumping to a vacuum of 5 × 10^−5^, after which the entire chamber was flushed with argon and pumped down again. After several chamber flushing processes, the alloy was prepared by melting all the elements several times to homogenize them in the entire volume. Finally, the liquid alloy was sucked into a specially prepared mold, which made it possible to obtain rods with a diameter of 3 mm and a length of 150 mm. The material prepared in this way was then subjected to mechanical treatment in order to be able to place it in devices for energy harvesting.

### 2.2. Ferro Magnetic Generator (FMG)

The tests performed in this work are mainly based on the principles of Ferro Magnetic Generators (FMG). As is generally known, the working principle is based on ferromagnets that are demagnetized either by shock loading or from the impact of high-speed flyers. The same effect might also occur as a result of the detonation of high explosives. The team at the Agency for Defense Development in Korea presented the output characteristics of annulus- and cylinder-type explosion-driven devices based on NdFeB magnets [23]. In this paper, the authors decided to use similar principles to FMG to obtain energy that could possibly be used to supply microcontrollers, but without the use of an explosion-driven device, and only with a force impulse. The force impulse causes a change in the magnetization around the material, then this change in the magnetic field is picked up by the coil and converted into electric energy.

In the case of a magnet, during an impact, it will be demagnetized (partially), and with a sufficiently large number of impacts, it could be fully demagnetized. In addition, in the event of an impact with sufficiently high energy, it could even be destroyed, generating a high amount of energy. On the other hand, the results showed that the use of magnets alone as a source of energy may be insufficient, as can be seen from the results shown in this paper.

## 3. Results

This section is divided into appropriate subsections, allowing the presentation of the course of the processes and the evolution of subsequent solutions, leading to the achievement of the aims of the study. The first part of this section describes the devices for energy harvesting that were then used in the experimental activities described in the second part.

### 3.1. Harvesters

**Figure 1:**
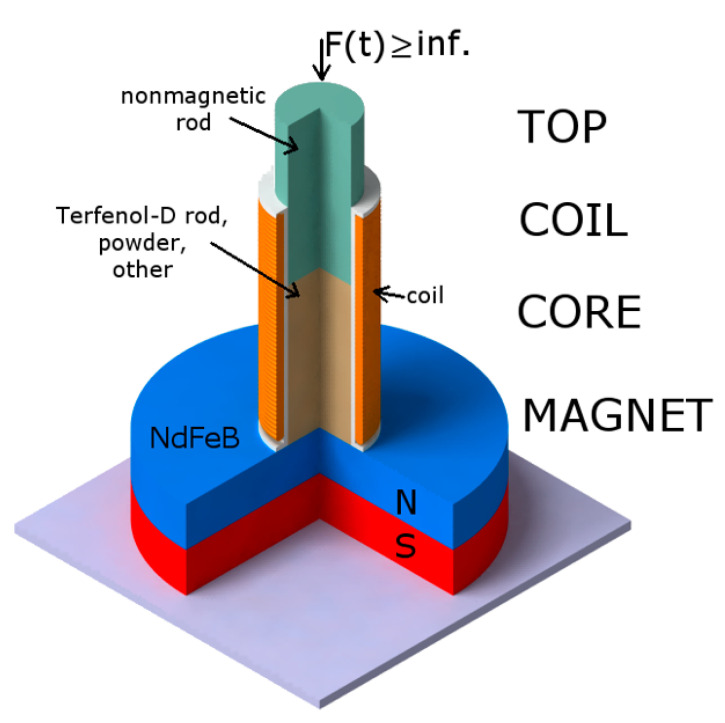
TCCM harvester scheme.

The research object is a unique basic Energy Harvester Device (EHD) model, shown in Figure 1. The model, called TCCM (Top Core Coil Magnet), is a construction consisting of four major elements: the Top, the role of which is to transfer the shock to the core, and the Coil, Magnet, and Core, which process the impact energy passed from the top, transforming it into electricity. For the aforementioned shock, a reference shock with specific repeatable parameters was used, thanks to which it was possible to test the harvester cores under the same working conditions. However, it should be noted that these devices can also operate in the case of various oscillating systems (with vibrations) of appropriate amplitude.

- A permanent NdFeB was used as a magnet.
- The material used for the core was important, because this is the part of the device responsible for the generation of current of sufficient value to operate as a pulse power supply. A few different core arrangements are proposed. The main material used was Terfenol-D and its powder. Additionally, pieces of the newly produced alloy were introduced to the core arrangement.
- The coil with a plastic body (the number of turns was 980, the resistance was 14Ohm, and the inductance was 1.1 mH) was fixed to the neodymium magnet.

As can be seen from the cross-section (Figure 1), TCCM harvesters can be considered to be one of the simplest models, especially since they do not have a system allowing the introduction of prestress, which makes it a structure with relatively low stiffness, especially when the force impulse is given. It should be noted that due to the simplicity of this structure, the device allows a large number of different materials to be tested relatively quickly for suitability in the field of energy recovery.

**Figure 2:**
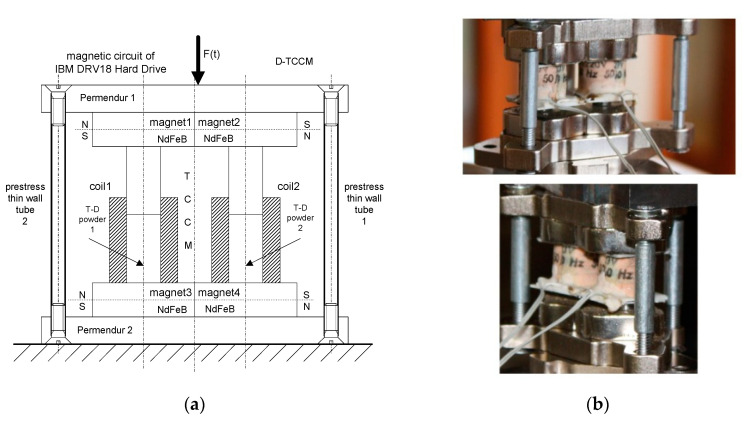
DTCCM harvester: (**a**) scheme, (**b**) real device.

The construction of a more advanced version of TCCM called Double Top Core Coil Magnet (DTCCM) is shown in Figure 2. This is a modified version of the TCCM harvester in which two magnetoelectric circuits are applied. The magnetic circuit of this device is also based on neodymium magnets, and it has low dispersion of magnetic field on the outside due to the permendur plates.

- Two coils used in the shown construction are the same, and have the same parameters, with static resistance R_c_ = 11 kΩ.
- In this type of harvester, it is possible to apply and control prestress. This is due to the thin-walled steel tubes, in which screw-bolts are placed, but the strength of both the screws and the tubes acts as a limitation.

Additionally, different arrangements of cores were also used.

### 3.2. Test Stand and Measurements

**Figure 3:**
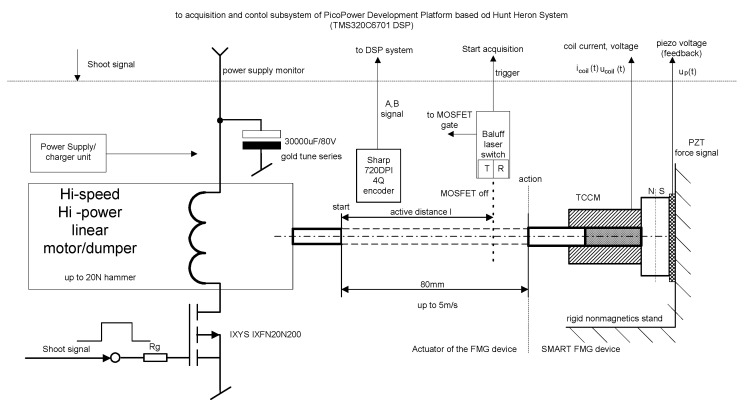
Test stand for TCCM harvester investigations.

Figure 3 shows the scheme of the stand developed to perform impact tests for energy harvesting devices, in particular, TCCM devices. A horizontally mounted PZT sensor was used to measure the impact force. The sensor was placed on a rigid, non-deformable surface of non-magnetic plate. To generate the impulse force in the device, an aluminum rod was used, which was mounted so that it always hit the center of the upper part of the device. The velocity of the rod movement, and thus the value of the impact force, was regulated. A fast MOSFET transistor interacting with a linear motor was used for regulation, which made it possible to set a precisely defined velocity. Additionally, the impact energy can be changed by changing the load applied to the moving element using weights of a known mass. The mass of each weight was determined with an accuracy of four decimal places. A maximum of 2 kg can be placed on the movable element, but it was decided to apply 0.5 kg due to the small size of the part with the aluminum rod. Thanks to the applied solutions, it was possible to obtain repeatable values of impact energy E_k_.

**Figure 4:**
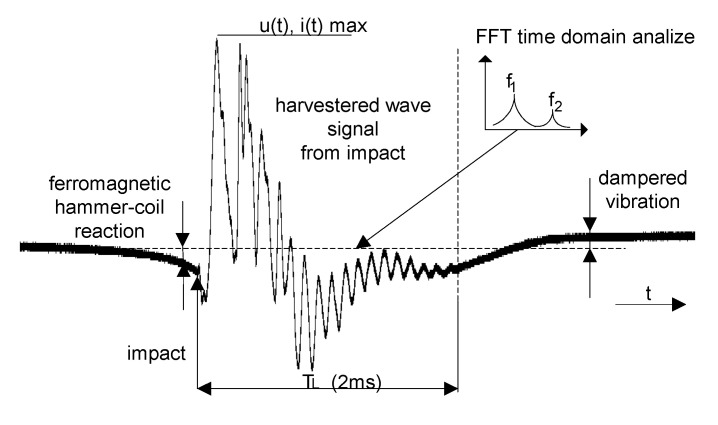
Example of a waveform obtained from TCCM.

The prepared system made it possible to test the cores placed inside the coil of the TCCM device. The impulse force resulting from the movement of the hammer in the form of the aluminum rod is transmitted to the core of the device through its upper part (Top). Figure 4 shows the response of the system during the experiment. Performing the analysis with respect to the experimental process, it is possible to specify individual phases within the experiment. The first phase is the phase just before it hits the device, where the voltage can be observed to rise. This change in voltage is probably caused by the movement of the ram in the magnetic field of the applied neodymium magnet. The next phase is the impact, which causes a shock wave to go through the consecutive elements of the harvester, to the magnet located at the bottom. This changes the magnetic flux, thus inducing a voltage on the used coil.

In the case of the TCCM harvester, only the top and the core of the system affect the resonance frequency of the system. The shock wave generated by the impact can circulate in the core–magnet system until it is completely suppressed, or exit the system through a magnet that is in contact with another rigid surface. However, it should be noted that this wave is not transferred to the coil itself. It can be observed that the mechanical resonance of the device resulting from the impact is a decisive factor for the signal obtained from the device.

In the case of the energy harvesting device used in the experiment, it was found that the prestress system did not need to be used. It was observed that the prestress effect was obtained during the first moments of impact. This was also the moment at which the maximum response values of the tested system were obtained. It should be noted that the values of the generated voltage were dependent on the applied electromagnetic system, which was in the form of a coil. When applying the same impact energy, the obtained voltage values, apart from the materials used for the core of the device, were also dependent on the number of turns in the coil.

The main goal of this investigation was to show the differences between the materials used to produce the core of the harvester in terms of the current values obtained for each of them, as well as with respect to the reaction of the PZT sensor and TCCM for impact. The final research step was to perform the core selection, which is a significant element of the harvesting device.

The original current signals from the coil were windowed using the Hamming windowing function. All current measurements were performed using a sampling rate of 1 MHz. In line with this, FFT analysis was performed for a spectrum of 500 kHz. Due to the applied windowing, the analysis of the spectrum was narrowed to 5 kHz and 10 kHz when using Terfenol-D as the core material.

**Figure 5:**
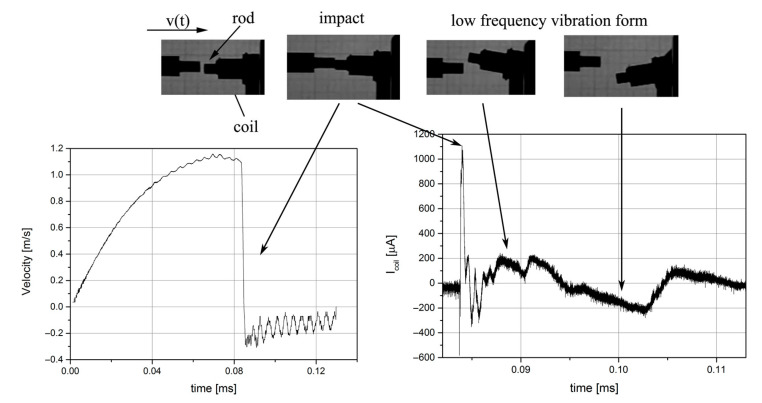
Impact correlated with graphs of velocity and output voltage.

As an output of the impact (the applied shock force), the velocity and voltage were determined, and their values are presented in Figure 5, where a correlation between the output signals and the actual test phases is presented.

The first phase of the test encompassed the acceleration of the aluminum ram up to a velocity of 1.1 m/s, E_k_ = 1.21 J, as shown in Figure 5. The second phase was the moment of impact, upon which the ram rapidly slowed down, and its reflection moment was controlled and damped. In the worst scenario, when the ram was not aligned with the harvester axis, vibration occurred throughout the whole device. On the graphs in Figure 5, the moment of impact is visible. A rapid increase in the current value appears at the moment of impact.

**Figure 6:**
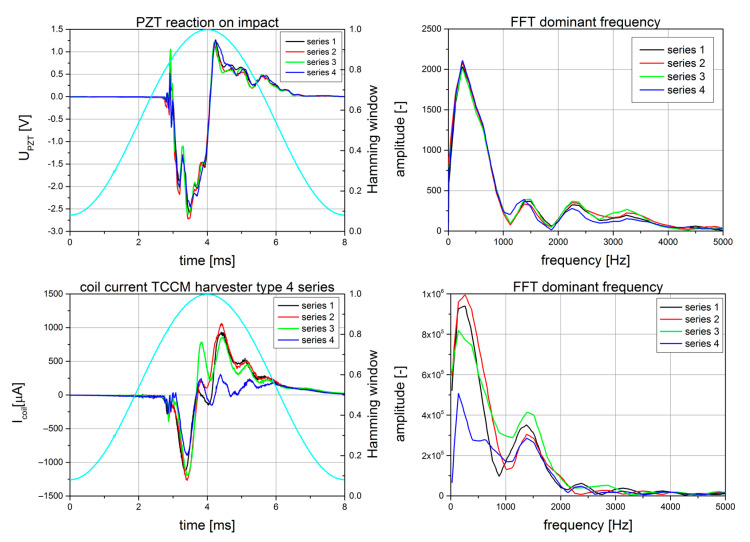
Reaction on impact: the PZT sensor signal, current of coil for TCCM, and their FFT spectrums.

In Figure 6, the results of an experiment with a series of four impacts with the same parameters and a TCCM harvester are presented. Analysis of the signal from the PZT piezo patch revealed the nature of the impact, as well as the reaction of the surface to which the harvester was fixed. However, according to the fact that loading the PZT with an impedance value of 1 MΩ decreases the surface signal one hundred times to the order of a few µV, the practical influence of that signal is negligible. By registering the signal from the PZT, it was possible to control the repeatability of the shock tests, except that, in the future, it will be possible to use that signal as a coupling to change the stiffness of the next generation of harvesters, with the intention of absorbing the maximum amount of energy from the impact with minimal transmission of that energy to the surface. However, the repeatability of the signal in the PZT did not have a place in the current signal of the coil. The reverse magnetostriction effect (Villari’s effect), and ensuring that the system has the appropriate operating parameters are very important. Even small changes in the impact conditions, mainly differences in the ram or the core, resulted in significant changes in the current values obtained. The operating parameters of the system are also greatly influenced by the external bias field, the pre-stress, and the impact energy.

It was important to assess the influence of the state of aggregation (solid, powder) of the core material on the output values. The main criterion for analyzing this influence was the absolute value (the ideal looseness straightens the AC signal) of the current during the 1 ms following the impact moment.

The fundamental frequencies of mechanical resonance are the second parameter describing the core composition. The coil resistive load value R_c_ = 125 Ω was determined using the math library package Numerix SIGLIB v6.0, implemented in the Agilent VEE Pro software.

During the impact tests, the couple–core–magnet system reacts rapidly, while the coil’s body is not expanded by the core material. When the impact energy becomes critical, E_k_ max, an irreversible change in system parameters occurs, resulting in the deformation of the coil body. This becomes an important issue when the core is in a powder state, because the powder adapts itself to the shape of the coil.

**Figure 7:**
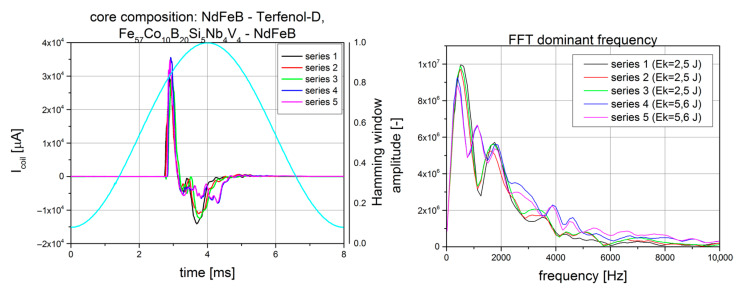
Example of the coil current measured for solid Terfenol-D with Fe_57_Co_10_B_20_Si_5_Nb_4_V_4_ alloy pieces, as well as its FFT.

During the investigation, it could be seen that the core material had a crucial influence on the results of the tests. These results are shown in Table 1, where the differences between four measured parameters can be seen. It can be observed that the highest value of I_max_ was obtained for the Terefenol-D rod with small pieces of the prepared Fe_57_Co_10_B_20_Si_5_Nb_4_V_4_ alloy used as the core. However, due to the high cost of Terfenol-D and its brittleness, the number of tests was limited. As an alternative for a solid rod made using that material, a powder form that was capable of withstanding impact energies greater than those achievable for steel and NdFeB was used. Additionally, the Terenol-D powder was partially mixed with powdered Fe_57_Co_10_B_20_Si_5_Nb_4_V_4_ alloy. The results of the impact tests for the powder core are shown in Figure 7. The only limitations of the experiment were the performance of the body and the durability of the coil windings. However, these two parameters were taken under consideration.

Where:

- Total_Load_ = 125 Ω is the resistive load on the coil, which is a source of generated energy due to an impact.
- R_c_ = 180 Ω is the static coil resistance (in this case quite a high value). In the case of harvesting energy from the reverse magnetostrictive effect, the coil parameters, i.e., how many Ohms and how many turns the coil has, are an important factor. In this paper, the aim was to generate the highest possible voltage increase so that the microprocessor power rectifier system was able to work under optimal conditions.
- I_max_ is the parameter that indicates the ability to power the microprocessor system from a single hit. This is the maximum current obtained during the impact phenomenon and the frequency response of the natural vibrations of the harvester’s core. In the case of using magnetostrictive cores, the I_max_ parameter is two orders higher than in the case of using other materials.

The test performed using powdered Terfenol-D mixed with Fe_57_Co_10_B_20_Si_5_Nb_4_V_4_ alloy as the harvester core, even without to the application of prestress, showed that with this type of core, it was possible to achieve higher results for the investigated parameters than when using the other listed materials. The lowest results were obtained for the sintered ferrite-type core.

**Figure 8:**
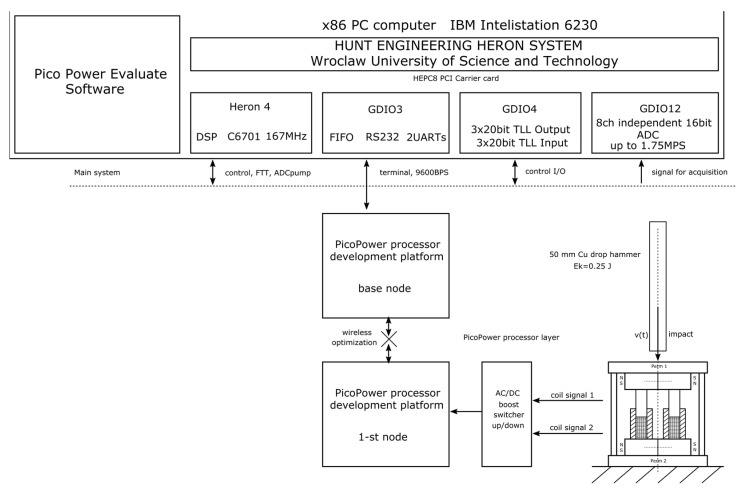
The PicoPower Development Platform was applied as a system to construct a new type of harvesting power supply.

The tests carried out using the TCCM devices made it possible to perform a comparison of the different types of materials used for the harvester core. However, it should be noted that regardless of the material used, the amount of energy obtained from the device was insufficient to power the microcontroller. Therefore, in the next step, it was decided to modify the test stand to carry out tests with the DTCCM harvester type. For this purpose, the PicoPower platform was specifically developed by the authors, based on the Hereon Hunt system. The block diagram for the research stand is shown in Figure 8. The data acquisition system that was used played a significant role in the stand. This system made it possible to obtain data with a very low level of noise in real time from all sources plugged into it.

The main component of data transfer between the Heron DSP chip and the host was one FIFO buffer that was connected to the PCI interface on the motherboard. The HEDG12 module was used to acquire the coil parameters in the form of current and voltage. This module is a 16-bit ADC converter with eight channels. Thanks to the use of this module, it was possible to acquire data on all eight channels using sigma-delta conversion.

The sigma-delta conversion technique used made it possible to eliminate analog anti-aliasing filters from the measurement path. Simply put, this technique oversamples the waveform that appears at the input eight times. The next step is to digitally filter the over-sampled data. Finally, the resulting samples are reduced eightfold.

**Figure 9:**
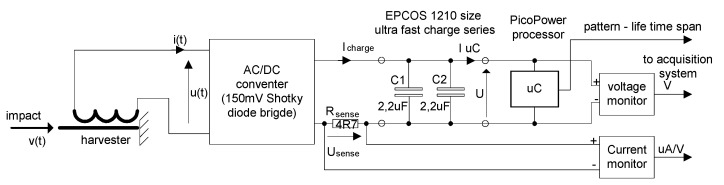
Supply current and voltage measurement scheme for detecting the lifetime of a powered microcontroller.

The devices presented in this paper are not intended to serve as continuous energy sources for microcontrollers. The main purpose of the developed harvesting devices is to provide a sufficiently large energy pulse to be able to charge a large-capacity capacitor. However, this does not mean that they cannot act as such a source of energy. For the devices shown in the paper, the manner in which they work will depend on the system specification. If the system is intended to send signals every few milliseconds, then the amount of energy supplied may not be large enough to accomplish such tasks. However, if the signal is to be sent every few hours, days, etc., then such a system could be regarded as a continuous source of energy. Figure 9 shows the principle of the system’s operation. Additionally, the capacitors would have to act fast enough to capture the current impulse in tens of μm.

**Figure 10:**
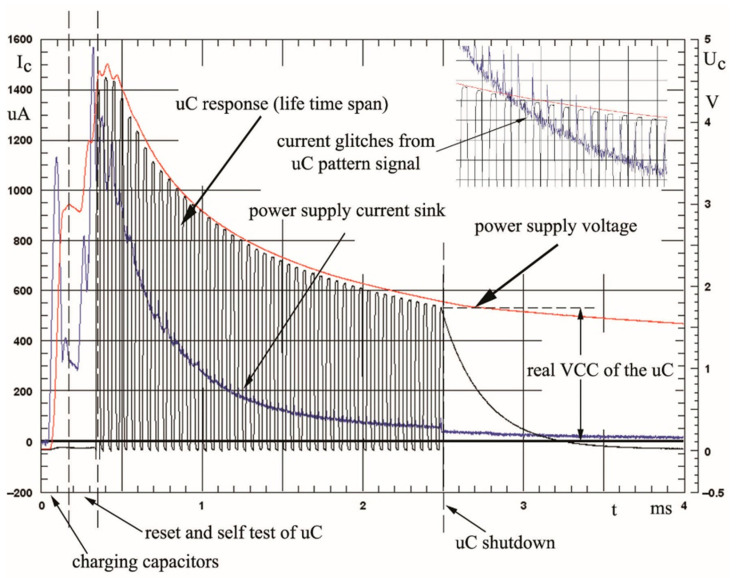
The lifetime of the ATMEGA48V microcontroller circuit powered by the DTCCM harvesting device at a low impact energy of E_k_ = 0.25 J.

Figure 10 shows the implementation of the developed energy harvesting device in practice, i.e., with the use of a microcontroller. The voltage drop that can be noticed on the measuring resistor, R_sense_ = 4.7 (Figure 9), can be read as the current consumed by the capacitor–microcontroller system. Because the capacitors C1 and C2 (Figure 9) are charged in the first phase, a significant increase in current can be noticed. These capacitors represent a large load for the developed harvester. In the moment at which current consumption decreased, the voltage reached a value of U_c_ = 3 V (Figure 9), making it possible to initiate microcontroller operation. In the first step, the microcontroller performed a reset, and then started to implement the saved algorithm. The microcontroller was supposed to wait until the capacitors were charged to about 5 V before performing the next actions. In the moment at which this value was reached, the microcontroller was to start generating signal sequences, which were then counted and forwarded by the RS232 HEGD3 module.

The lifetime algorithm of the program worked from U_max_ = 5 V to U_min_ = 1.8 V. During this voltage drop, the microprocessor was able to send about 50 pulses, giving a microcontroller life of about 3 ms. By selecting various mechanical excitation sources, values of up to 200 pulses were obtained, which extended the lifetime of the microcontroller to 8 ms. Finally, an Energy Harvesting Device platform was developed that was able to supply a popular microcontroller, realizing its code for a lifetime of 3 ms with a low impact energy of E_k_ = 0.25 J. The presented device was based on a core made of Terfenol-D powder mixed with Fe_57_Co_10_B_20_Si_5_Nb_4_V_4_ alloy powder.

In the case of the proposed system, it seems difficult to estimate its efficiency. It cannot be easily calculated, because the electrical efficiency of the system is dependent on the efficiency of each of its components, and the measurement path is complicated. It is obvious that the higher the efficiency of converting energy from one form to another, the better it will be. However, attention should be paid to the fact that the proposed solution needs to work in places where energy is not processed in any way, but only lost in the form of mechanical vibrations. In such cases, recovering or changing even a small part of the energy into another form of energy (in this case electric) seems to make the most sense, even though the efficiency of this transformation is not at a high level. The device shown in this work is based on the reverse magnetostriction effect, but of course, work is also underway using inverse pizoelectric effects, the Farraday effect, and electrostatic charges. In the case of the presented solution, the goal was to develop a device that would not have any moving parts, thus limiting the possibility of defects to some extent. In further research work, it is planned to modify both the measurement system and the harvester in order to increase the amount of electricity generated.

## 4. Conclusions

Two types of simple energy harvesting devices were developed and investigated using dedicated test stands. The first type of harvester device was used to perform basic research on the influence of the core type on the resulting values of electric energy. On the basis of this series of tests, it is possible to conclude that:

- The proper choice of the coil (the number of coils and its impedance) and the core material for the harvesting device is very important, and has a significant influence on the results.
- Providing low-frequency vibration might have significant implications for the practical realization of energy harvesting, and application in industrial objects, as it could support the optimal operation (generation of current) of the harvester for a long time. However, it should be noted that the value of current generated as a result of impacts will be much higher than that generated from vibrations.
- In the coil and PZT sensor, the same frequencies were dominant, although the PZT spectrum had further harmonics that did not occur in the current signal of the coil. It was found that the two frequencies characterizing the material of the harvester core are sufficient.
- In the low-energy range of impulses, Ek ≤ 5.6 J, the application of Terfenol-D rod as the core results in the device to being able to supply about ten 8-bit RISC microelements in a time of about 1 ms.
- To ensure optimal prestress, while also increasing the resonance frequency and the lifetime of waves passing through the harvester device, the TCCM structure was modified. The developed DTCCM harvester structure made it possible to apply and tune the prestress value to the core of the device.
- Finally, an Energy Harvesting Device platform was developed that was able to supply a popular microcontroller that realizes its code within a timeframe of 3 ms with a low impact energy of Ek = 0.25 J.

Moreover, the results obtained in the presented work may constitute a base for further application work with the developed materials, particularly in the field of energy harvesting.

The solution proposed in this paper may find application in various areas, including the transport and manufacturing industries. These devices could be placed under or in the vicinity of railway tracks or on railway carriages. Another example would be trucks or loading bases, where devices could be placed in the ground (e.g., around speed bumps). In addition, devices of this type could find application in the manufacturing industry, especially in steel mills and pressing plants, near presses, or hammers. Another interesting application is the use of the proposed device in ballistics equipment. As additional work, cooperation has been initiated with a group of scientists from the Department of Mechanics, Mechanical and Biomedical Engineering, researching ballistic shields. When testing such shields, large amounts of energy are generated and lost at the same time. Work is currently underway on the effective use of the proposed devices to recover at least a small part of the energy supplied to ballistic shields.

## Figures and Tables

**Table 1:** Comparison of different harvesting cores with parameters as follows: Total_Load_ = 125 Ω, E_k_ = 5.6 J, R_c_ = 180 Ω for V = 6.7 m/s, and m = 0.5 kg.

Core Type	I_max_ [μA]	ABS I_max_ (1 ms) [μA]	FFT Dominate Frequency	
			F1 [Hz]	F2 [Hz]
Cu	1200	600	375	1500
Fe	1600	500	500	1750
Ferrite	500	200	375	1500
NdFeB	1200	400	875	1500
Terfenol-D powder mixed with Fe_57_Co_10_B_20_Si_5_Nb_4_V_4_	2500	800	250	1625
Terfenol-D rod with Fe_57_Co_10_B_20_Si_5_Nb_4_V_4_ pieces	35,000	8300	500	1875

## Data Availability

Data supporting reported results can be provided upon request. Currently, these data are collected as part of the ongoing project, and only after its completion will the data be made available to the public.
